# Tumor Metabolism of Malignant Gliomas

**DOI:** 10.3390/cancers5041469

**Published:** 2013-11-08

**Authors:** Peng Ru, Terence M. Williams, Arnab Chakravarti, Deliang Guo

**Affiliations:** Department of Radiation Oncology, Ohio State University Comprehensive Cancer Center & Arthur G James Cancer Hospital, Columbus, OH 43012, USA; E-Mails: peng.ru@osumc.edu (P.R.); terence.williams@osumc.edu, (T.M.W.); arnab.chakravarti@osumc.edu (A.C.)

**Keywords:** glioblastoma, tumor metabolism, SREBP-1, LDLR, LXR, glucose, lipids, cholesterol

## Abstract

Constitutively activated oncogenic signaling via genetic mutations such as in the EGFR/PI3K/Akt and Ras/RAF/MEK pathways has been recognized as a major driver for tumorigenesis in most cancers. Recent insights into tumor metabolism have further revealed that oncogenic signaling pathways directly promote metabolic reprogramming to upregulate biosynthesis of lipids, carbohydrates, protein, DNA and RNA, leading to enhanced growth of human tumors. Therefore, targeting cell metabolism has become a novel direction for drug development in oncology. In malignant gliomas, metabolism pathways of glucose, glutamine and lipid are significantly reprogrammed. Moreover, molecular mechanisms causing these metabolic changes are just starting to be unraveled. In this review, we will summarize recent studies revealing critical gene alterations that lead to metabolic changes in malignant gliomas, and also discuss promising therapeutic strategies via targeting the key players in metabolic regulation.

## 1. Introduction

Malignant glioblastomas (GBM) are aggressive brain tumors, which are resistant to radiation and chemotherapy [1,2,3,4]. In the last decade, targeted inhibition of oncogenic signaling pathways such as EGFR [5,6,7,8,9], PI3K/Akt [10,11,12,13], and VEGF [14] has made some progress. However, in patients with malignant glioma, these treatments only show a transient effect and tumor cells quickly develop resistance [15,16]. Therefore, in order to significantly improve patient survival, it is necessary to fully understand the biology of malignant gliomas.

**Figure 1:**
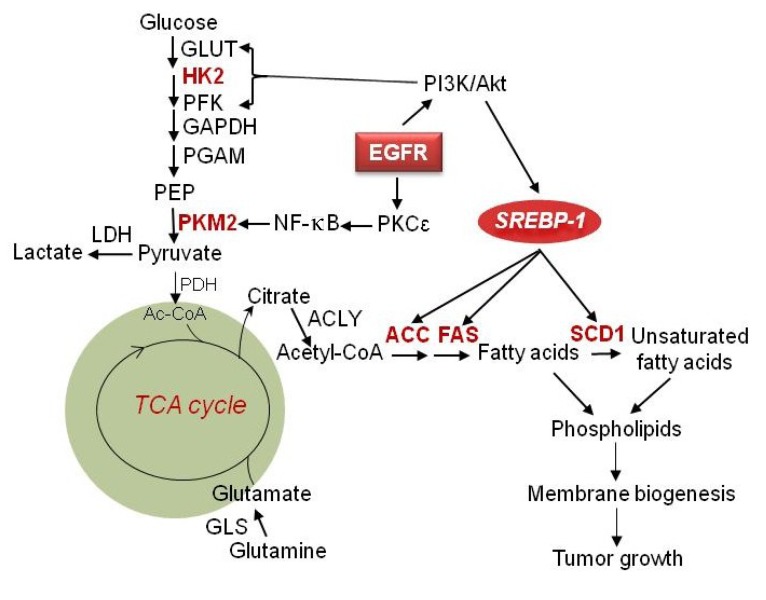
Regulation of metabolism in maligant gliomas. EGFR signaling upregulates glycolysis and is mediated by PI3K/Akt signaling through inducing GLUT1 translocation to the cell membrane as well as activation of HK2 and PFK. EGFR signaling also upregulates PKM2 expression via activation of the PKCε/NF-κB signaling pathway. Large amounts of lactate are generated even in abundant oxygen conditions. In addition to oxidative phosphorylation, glucose is converted to fatty acids through generation of citrate in the mitochondria, which is in turn released into to cytoplasm and lysed by ACLY to generate the fatty acid precursor acetyl-CoA. Acetyl-CoA is then used for palmitate synthesis and is regulated by ACC and FAS. SCD1 further catalyzes saturated fatty acids to monounsaturated fatty acids. Together they form phospholipids for membrane formation and promote tumor growth. EGFR/PI3K/Akt signaling also activates SREBP-1, which upregulates ACC, FAS and SCD1, to promote *de novo* fatty acid synthesis. Furthermore, glutamine enters into the TCA cycle to generate energy and also can be converted to lipids through reductive carboxylation under hypoxic conditions. GLUT, glucose transporter; HK2, hexokinase 2; PFK, phosphofructokinase; GAPDH, glyceraldehyde 3-phosphate dehydrogenase; PGAM, phosphoglycerate mutase; PEP, phosphoenolpyruvic acid; PKM2, pyruvate kinase M2; LDH, lactate dehydrogenase; PDH, pyruvate dehydrogenase; TCA cycle, tricarboxylic acid cycle; ACLY, ATP citrate lyase; ACC, acetyl-CoA carboxylase; FAS, fatty acid synthase; GLS, glutaminase; EGFR, epidermal growth factor receptor; SREBP-1, sterol regulatory element-binding protein 1.

In recent years, the understanding of the regulation of tumor metabolism has significantly improved. Accumulating evidence show that tumor cells reprogram their metabolism to meet high energy demands, coordinate markedly elevated biosynthetic processes and energy production, which in turn promote rapid growth and division of tumor cells [17,18,19,20]. Thus, targeting metabolism has become a novel promising strategy for treating cancers. Recently, enhanced glycolysis [21,22], elevated glutaminolysis [10,23], and exacerbated lipogenesis have been demonstrated as prominent characteristics in glioblastoma (GBM) [24,25,26,27,28,29]. There are several important regulators of metabolic pathways, such as Hexokinase 2 (HK2), PKM2 [30,31], IDH1 [32], SREBP-1 and LDLR [24,25,28,29], that have been revealed to be upregulated in GBM. EGFR/PI3K/Akt signaling has been shown to be involved in the regulation of lipid metabolism in GBM [24,25,28,29] (Figure 1). Targeting SREBP-1, a key regulator in lipid metabolism (Figure 1), significantly inhibited tumor cell growth in GBM cell lines and xenograft models [28,33]. 

In this review, we will focus on the basic metabolism pathways in GBM including glucose, glutamine, and lipid metabolism, and we will discuss the reprogramming of metabolic pathways and the molecular link between oncogenic signaling pathways and key metabolic regulators.

## 2. Glucose Metabolism in GBM

In normal tissues, ATP is primarily generated in mitochondria via complete oxidative phosphorylation (OXPHOS) of glucose. Conversely, only 10% of ATP is generated from glycolysis in which glucose is converted to lactate [34]. Intriguingly, tumor tissues demonstrate high levels of glycolysis even under aerobic conditions, which is upregulated by PI3K/Akt signaling, known as the Warburg effect [35,36,37,38,39,40] (Figure 1). In malignant gliomas, aerobic glycolysis is one of the major characteristics of these tumors [21,41]. A number of metabolic enzymes, such as HK2, PKM2 and IDH, play a crucial role in glucose metabolism, and serve as attractive molecular targets.

### 2.1. Hexokinase II

Hexokinase (HK) functions in the first step of glycolysis and irreversibly catalyzes the phosphorylation of glucose to glucose-6 phosphate (G6P) [42] (Figure 1). Hexokinase has four isoforms referred as type I, II, III and IV which are identified from different mammalian tissues [43]. The type I, II and III HKs have molecular weight at approximately 100 kilodaltons (KD) and can be inhibited by their product G6P. Type IV HK, also known as glucokinase (GK), displays different kinetics from other HK isoforms with a molecular weight of 50 KD [44,45]. In GBM, HK2 is highly expressed, whereas HK1 is predominantly expressed in normal brain and low-grade gliomas [46]. Interestingly, HK2 is expressed in lower levels in the neural subtype, but at higher levels in mesenchymal subtype of GBM [46,47]. In addition, increased HK2 gene expression is correlated with poor overall survival in GBM patients [46,48]. Overexpression of HK2 in GBM promotes tumor cells proliferation, and enhances their resistance to radiation and temozolomide treatment. In contrast, knockdown of HK2 but not HK1 decreases cell proliferation and enhances therapeutic sensitivity in GBM cell line and xenograft models [46].

### 2.2. PKM2

PKM2 is the M2 isoform of pyruvate kinase (PK) [49]. PK functions in the last step of glycolysis, catalyzing phosphoenolpyruvate (PEP) to pyruvate and generating ATP [49]. Four PK isoforms are identified and derived from two genes, *PKLR* and *PKM* in mammals. R and L isoforms are derived from *PKLR*, expressed in erythrocytes and the liver [49]. M1 and M2 isoforms of PK are produced by alternative splicing of *PKM* gene, *PKM1* contains exon 9 and *PKM2* contains exon 10, respectively [50]. Exon 9 and 10 of *PKM* show different properties, which results in the different activity of PKM1 and PKM2. PKM1 is constitutively active, but PKM2 can be regulated by fructose-1,6-bisphosphate. Recently, splicing repressors of hnRNP A1, hnRNP A2 (heterogeneous nuclear ribonucleoprotein, hnRNP) and PTB (polypyrimidine tract binding protein) were reported to directly bind to the flanking intron of exon 9 and repress the use of exon 9. Downregulation of these splicing repressors increased PKM1 expression levels in cancer cells [48].

**Figure 2:**
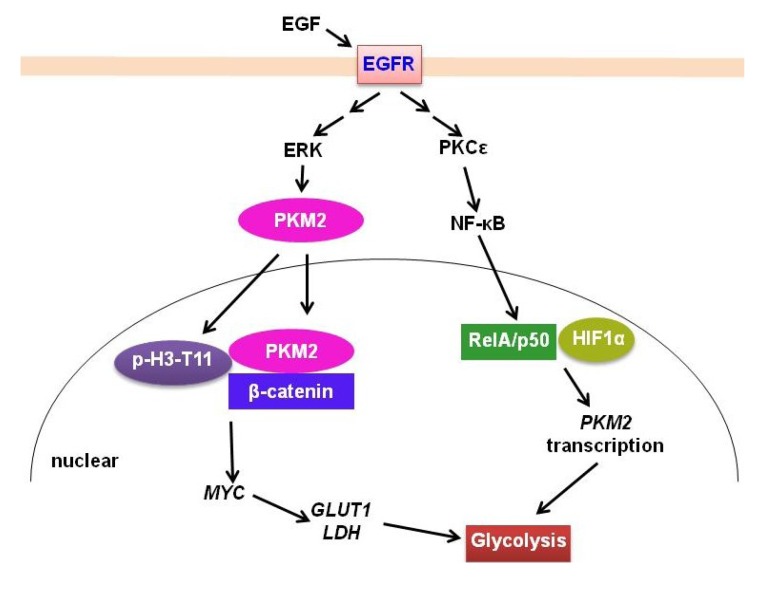
EGFR signaling regulation of PKM2 in gliomas. EGFR signaling promotes PKM2 translocation to nucleus after phosphorylation by ERK. PKM2 then phosphorylates histone H3-T11 and binds to β-catenin to promote MYC expression. MYC stimulates GLUT1 and LDH expression to further promote glycolysis. Moreover, EGFR signaling upregulates PKM2 expression, which is mediated by PKCε/NF-κB activation and interaction with HIF1 α.

PKM2 is mainly expressed in proliferating cells and tissues. In cancer, PKM2, not PKM1, is the major isoform expressed [49,51,52]. Recently, PKM2 has been revealed to be involved in the EGFR signaling pathway in GBM [31,53,54,55] (Figure 1). PKM2 was shown to translocate into the nucleus from the cytoplasm when activated by EGFR [55]. PKM2, not PKM1, is phosphorylated at Ser37 by ERK1/2 after EGF stimulation. The phosphorylated PKM2 recruits protein interacting with NIMA (Never in Mitosis A)-1 (PIN1) and binds to importin α5, which results in the translocation of PKM2 from cytoplasm to the nucleus. After entering into the nucleus, PKM2 phosphorylates histone 3 at T7 site and also acts as a co-activator of β-catenin to then promote the transcription of *CCND1* and *MYC*. C-Myc further upregulates glucose transporter (GLUT) and HK2 to promote glycolysis (Figure 2) [54]. Interestingly, GLUT3 has been shown to be highly express in gliomas correlated with tumors grade [56,57]. Moreover, in GBM cells, EGFR signaling was shown to also upregulate the expression of *PKM2*. Activation of EGFR by EGF induces PKCε monoubiquitylation, which then recruits and phosphorylates IKKβ, to further promote the interaction of activated RelA/p50 with HIF1α. This complex then binds to the promoter of *PKM2* and promotes its transcription (Figure 2) [31]. Recently, miR-326 has been reported to inhibit GBM by directly targeting PKM2 [30]. Taken together, these data demonstrated the novel roles for PKM2 in GBM beyond its traditional roles in glycolysis.

### 2.3. IDH

Isocitrate dehydrogenase (IDH) is an enzyme that catalyzes the oxidative decarboxylation of isocitrate to produce α-ketoglutarate, and also generate NADH in mitochondria or NADPH in cytoplasm [58]. In the human genome, 5 IDH genes have been identified: two of them produce IDH1 and IDH2, which form homodimers; the other 3 genes produce IDH3 (IDH3α, IDH3β, and IDH3γ), which form heterotetrameric proteins [59]. IDH1 is located in the cytoplasm and peroxisomes, but IDH2 and IDH3 are localized in the mitochondria [59].

In 2008, IDH1 and IDH2 mutations were first reported in low grade of gliomas and secondary GBM [60,61,62]. IDH1 mutation is described in more than 70% and 50% of grade II and III glioma tumors [63], but only 5% in primary GBM [64]. In addition, IDH1 mutations occur approximately in 80% and 85% of diffuse astrocytomas and secondary GBMs, respectively [59]. IDH1/2 mutation often co-occurs with the deletion of 1p19q and mutation of p53 [64,65]. IDH1 and IDH2 mutation results in reduced enzymatic ability and therefore reduces the production of α-KG [66]. α-KG is required by propyl hydroxylases (PHD) for degradation of hypoxia-induced factor-1α (HIF-1α) [67]. Therefore, IDH1 is involved in the HIF-1α signaling pathway, which is critical for tumor progression, glucose metabolism, angiogenesis, and invasion under low oxygen levels [68]. Surprisingly, IDH1/2 mutants gain new enzyme activity, which catalyzes α-KG to 2-hydroxyglutarate (2-HG), a metabolite produced mostly in error in normal metabolism where it is usually present in low levels [58,69,70,71]. In glioma patients, IDH mutations are associated with elevated 2-HG levels [69,70,71,72].

Recently, CpG island methylation profiling was performed to identify the difference between IDH1-R132 mutant and IDH1-R132 wild type GBM. The profiling analysis showed a similar methylation pattern in IDH1-R132 mutant GBM and a newly-described CpG island methylator phenotype (CIMP) [73]. The phenotypes of induction of extensive DNA hypermethylation with inhibition of histone demethylation were observed when the mutant IDH1 was introduced into primary human astrocytes. These data demonstrate that the mutation of IDH1 is sufficient to remodel the methylome in glioma and establish CIMP [74]. IDH mutation also reduces histone demethylation and inhibits differentiation of non-transformed astrocyte cells [75]. The expression of lineage-specific differentiation genes is inhibited by the introduction of mutant IDH1 or cell-permeable 2-HG [75]. The accumulation of histone methylation is observed with the introduction of 2-HG-producing mutant IDH into immortalized astrocytes [75]. In summary, these data strongly reveal that IDH mutations are clearly involved in the regulation of differentiation of non-transformed cells.

## 3. Glutamine Metabolism

In addition to glucose, glutamine is another major energy source for cells [76,77] (Figure 1). Glutamine also is a nitrogen donor for tumor cells [78]. Recently, glutamine metabolism has been reported to be highly upregulated in cancers [17,79,80,81,82]. Elevated expression of glutaminase (GLS) is found in tumors and rapidly proliferating cells [83] (Figure 1). GLS, located in the mitochondria, catalyzes the conversion of glutamine to glutamate, and is upregulated by the oncogene c-Myc [84,85]. NF-κB-dependent upregulation of glutaminase by oncogene Rho GTPase has also been described whereas blocking Rho GTPase leads to the inhibition of cancer growth [86]. Nuclear magnetic resonance (NMR) spectroscopy shows that glutamine concentration (3.4 ± 0.9 mmol/L) was significantly higher in GBM patients than in control subjects (2.7 ± 0.7 mmol/L; *p* = 0.01) [87]. Interestingly, lower expression of glutamine synthetase (GS), which catalyzes glutamate to glutamine correlates with better survival of patients with GBM [88]. Compared to our knowledge of glucose metabolism in cancers, glutamine metabolism in GBM is much less known. Thus significant investigations are needed to reveal the function of glutamine metabolism in GBM tumorigenesis and resistance to therapies.

## 4. Lipid Metabolism in GBM

Lipids consist of phospholipids, fatty acid, cholesterol, triglycerides, cholesterol esters, sphingolipids and others, which are critical components to form cellular membranes [89,90,91,92,93]. In addition to their role as structural components, lipids also function as energy resources and as signaling molecules to maintain cell growth [25,94,95].

**Figure 3:**
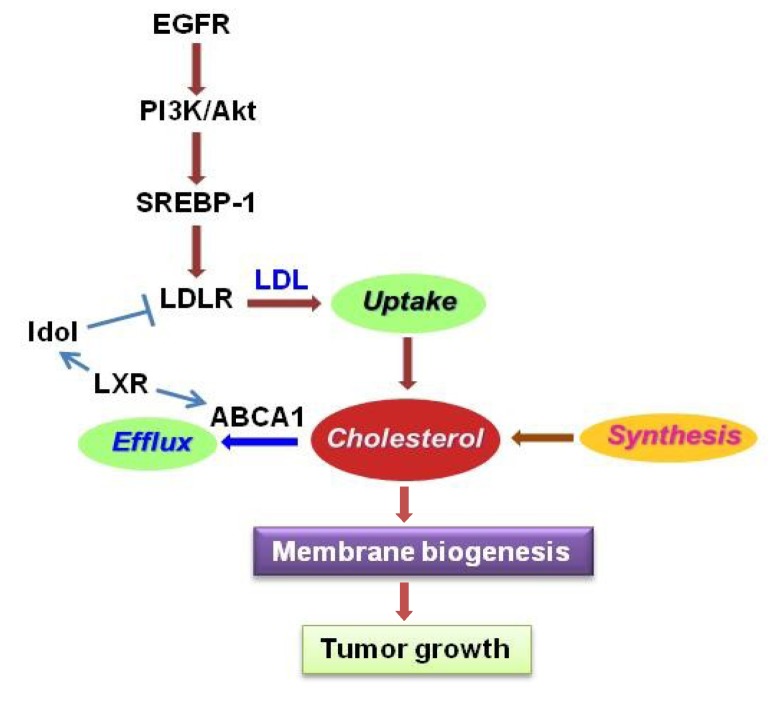
Regulation of cholesterol metabolism in gliomas. Cholesterol homeostasis is regulated by uptake, synthesis and efflux. EGFR/PI3K/Akt signaling upregulates LDLR expression mediated by SREBP-1 to promote cholesterol uptake. Activation of LXR stimulates ABCA1 expression and promotes cholesterol efflux, but also reduces LDLR levels via upregulation of Inducible Degrader of LDLR (Idol), a ubiquitin ligase E3. LXR, liver X receptor; ABCA1, ATP-binding cassette transporter member 1; LDL, low density lipoprotein; LDLR, LDL receptor.

Lipid metabolism has been found to be largely altered in cancers [96,97,98,99], and exacerbated lipogenesis has been shown to be a prominent characteristic in most cancers [25,94,100]. In GBM, tumor tissues contain higher levels of unsaturated fatty acids compared with normal brain [101]. Recent studies have revealed intrinsic molecular alterations in lipid metabolism. Key genes that regulate lipid metabolism, such as SREBP-1 and its downstream-targeted genes Acetyl-CoA carboxylase (ACC), Fatty acid synthase (FAS) and low-density lipoprotein receptor (LDLR), are upregulated in GBMs [27,28,29] (Figure 1 and Figure 3). Intriguingly, the oncogenic signaling pathway EGFR/PI3K/Akt has been shown to regulate this metabolic reprogramming [27,28,29] (Figure 1 and Figure 3). 

### 4.1. SREBP-1

Sterol regulatory element-binding proteins (SREBPs) are important transcription factors that regulate lipid metabolism thereby playing a crucial role in the regulation of lipogenesis and cholesterol uptake [102]. There are three SREBP isoforms, namely SREBP-1a, -1c and -2 [102]. SREBP-1 (-1a and -1c) regulates fatty acid synthesis [103] while SREBP-2 regulates cholesterol synthesis [104]. The transcriptional activity of SREBPs is tightly regulated by cellular sterol levels via post-translational regulation. Morevoer, the full length SREBP-1 or -2 proteins have no activity. Indeed, these proteins bind to SREBP cleavage-activating protein (SCAP) and are retained by Insig protein on the membrane of the endoplasmic reticulum (ER) when sterol levels are high [105]. However, when sterol levels decrease, SCAP will dissociate from Insig and then carry SREBPs to the Golgi [105]. Upon translocation to the Golgi, the *N*-terminus of SREBPs will be released from the membrane after sequential cleavage by the membrane-bound serine proteases, S1P and S2P [106,107]. Then, only the *N*-terminus enters into the nucleus to promote its downstream gene expression [104,108,109]. 

Interestingly, SREBP-1 has been found to be highly activated in GBM and other cancers [28,110,111,112,113]. Its nuclear form is abundantly present in GBM patient tissues, which is accompanied with the high expression of its downstream genes ACC and FAS, the key genes regulating *de novo* fatty acid synthesis [28] (Figure 1). Moreover, genetically or pharmaceutically inhibition of SREBP1, ACC or FAS in GBM has been shown to significantly induce cell death, suggesting that SREBP1 is a promising therapeutic target in GBM [28]. More recently, inhibition of SREBP-1 has been shown to markedly reduce the levels of unsaturated fatty acids by downregulating stearoyl-CoA desaturase 1 (SCD1), a key enzyme to catalyze saturated fatty acid to mono-unsaturated fatty acid (Figure 1), thus increasing the ratio of saturated- to unsaturated-fatty acids and leading to lipotoxicity in GBM cells [33,114].

In addition to the feedback regulation by sterol levels, SREBP-1 is highly upregulated by receptor tyrosine kinases (RTKs)/PI3K/Akt signaling in GBM [28] (Figure 1). More interestingly, inhibiting SREBP-1 and its downstream genes ACC and FAS has been shown to markedly induce cell death in GBM cells, particularly in cells with high levels of EGFR signaling [28,33]. These data demonstrate that GBM cells with activated RTK signaling are dependent on the SREBP-1-regulated *de novo* fatty acid synthesis pathway for survival and malignant growth.

### 4.2. LDLR

Cholesterol is also an important component of cell membranes [90,115]. In cells, extra cholesterol will be esterified with fatty acids to form cholesterol esters which are then stored within the cells [116]. Using NMR technique, cholesterol esters have been found to be abundantly present in tumor tissues in high grade gliomas, human urothelial carcinoma, and malignant renal cell carcinomas, but undetectable in normal tissues [116,117,118,119,120]. These findings demonstrate that cholesterol metabolism is also altered in these cancers. But the molecular mechanism underlying this alteration is largely unknown. 

Recently, low density lipoprotein receptor (LDLR) has been found to be upregulated in GBM patient tissues, xenograft tumors and cell lines, and this upregulation is correlated with high levels of cholesterol esters in GBM cells [29,121]. The function of LDLR is to bind low-density lipoprotein (LDL), the major cholesterol found in abundance within the bloodstream, and then bring LDL into cells and provide cholesterol for cell utilization [122]. Its upregulation could partially explain the accumulation of cholesterol esters in GBM. Interestingly, LDLR has been shown to also be upregulated by EGFR/PI3K/Akt signaling, which was been shown to be mediated by SREBP-1, but not SREBP-2 in GBM cells [29] (Figure 3). 

### 4.3. LXR/ABCA1

Cholesterol homeostasis is regulated by uptake, *de novo* synthesis, and efflux (Figure 3). When cellular cholesterol level increases, it will be oxygenized to oxidized sterol and then activates liver X receptor (LXR), a transcriptional factor that controls expression of cholesterol efflux genes, such as ATP-binding cassette protein A1 (ABCA1) and G1 (ABCG1) [123]. ABCA1 and ABCG1 promote reverse transport of cholesterol outside of cells, thus reducing cellular cholesterol levels [124] (Figure 3). Interestingly, activation of LXR by its agonists GW3965 and T0901317 has been shown to significantly inhibit tumor growth in GBM, breast, and prostate cancers [24,25,29,125]. Additionally, activation of LXR by GW3965 downregulates LDLR levels through upregulating a ubiquitin ligase E3 known as inducible degrader of LDLR (Idol), which leads to LDLR degradation [29,126] (Figure 3). Therefore, activation of LXR could be a potential therapeutic strategy to inhibit GBM growth through increasing cholesterol efflux as well as decreasing its uptake.

## 5. Conclusions

Metabolic reprogramming is a key feature driving oncogenesis in cancers. Recent studies have revealed that glucose, glutamine and lipid metabolism are largely altered in GBM and facilitate its malignant growth. Oncogenic signaling pathways are found to regulate these metabolic alterations. Targeting metabolic reprogramming is a novel and promising therapeutic strategy to treat cancers. Further understanding of the metabolic alterations in GBM will definitely shed light on developing effective approaches to abrogate GBM malignant growth, and also provide insights to overcome GBM resistance with current therapies. 
